# *Mycobacterium fortuitum* Infection in Breast Implant and Rib Osteomyelitis: A Case Report and Review of Literature

**DOI:** 10.1093/asjof/ojag119

**Published:** 2026-07-10

**Authors:** Paola Andrea Kafury Goeta, Mauricio Zuluaga Botero, Federico Reina Ramírez

## Abstract

Breast implant–associated *Mycobacterium fortuitum* infections with presumed rib osteomyelitis are exceedingly rare, with fewer than 7 cases reported globally. We report the first documented case from Latin America, identified through systematic review of PubMed and SciELO databases. A 25-year-old woman with no predisposing comorbidities underwent mastopexy and breast augmentation with a retropectoral silicone implant. She developed a persistent left breast wound unresponsive to conventional treatment at an outside institution and was referred to our center ∼14 months postoperatively with a chronic fistula and rib exposure. A multidisciplinary approach (plastic surgery, orthopedic surgery, and infectious disease) was employed. Serial surgical debridement with vacuum-assisted closure therapy was performed. Cultures confirmed *M. fortuitum* sensitive to clarithromycin and doxycycline; combination therapy with clarithromycin and moxifloxacin was administered for 6 months. Wound closure was achieved with epigastric and thoracoabdominal pedicle flaps, followed by bilateral autologous fat grafting for breast reconstruction. At 8-year follow-up, the patient reported satisfaction with the aesthetic result. This case emphasizes the importance of maintaining clinical suspicion for nontuberculous mycobacterial infection in chronic nonhealing wounds following breast implant surgery, even in immunocompetent patients without identifiable risk factors.

Level of Evidence: 5 (Therapeutic)

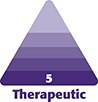

Breast implant infection occurs in 0.7% to 6% of cosmetic augmentations and up to 20% of reconstructive procedures.^1-4^ The most common causative organisms are *Staphylococcus aureus* and coagulase-negative staphylococci.^5,6^ Nontuberculous mycobacteria (NTM) are less frequent but increasingly reported agents; *Mycobacterium fortuitum*, a fast-growing mycobacterium, is particularly associated with hospital water environments and biofilm formation, making it resistant to standard disinfection.^7-9^ NTM infections are notoriously underdiagnosed because routine bacterial cultures are negative and clinical suspicion is rarely triggered.^9,10^ Rib osteomyelitis is an exceptionally rare complication of breast implant infection, with fewer than 7 cases previously reported, all from Europe and Australia.^11-15^ A systematic search of PubMed and SciELO databases confirmed no previous report from any Latin American country. To our knowledge, this is the first case in Latin America of *M. fortuitum* breast implant infection associated with presumed rib osteomyelitis.

## CASE PRESENTATION

Written informed consent was obtained from the patient for publication of this case report and accompanying images. The ethics committee approved this report (IRB letter CEI-482, Act No. 249), in accordance with Resolution 8430/1993 of the Colombian Ministry of Health, ICH-GCP guidelines, and the Declaration of Helsinki.

A 25-year-old woman with no relevant medical history, no drug allergies, and no preoperative medications underwent combined mastopexy and breast augmentation (325 cc textured silicone gel implant, retropectoral plane, periareolar incision with vertical technique), bilateral liposculpture, and cesarean scar revision at an outside institution. The early postoperative course was uneventful.

Approximately 4 months postoperatively, she developed burning sensation, pain, and increased left breast volume. Surgical exploration at the outside institution revealed a 150 cc seroma, which was drained with saline irrigation; the original implant was retained, and seroma fluid was not cultured. Three weeks later, persistent induration and erythema of the inferolateral pole prompted needle aspiration of a subcutaneous collection, yielding 3 cc of yellow fluid; the implant pocket was not directly accessed. Moxifloxacin was initiated orally, and the 7-day culture returned negative. Because of persistent secretion, the left implant was removed ∼5 months after the initial surgery. Throughout this period, the patient had no fever or systemic signs of infection.

Following implant removal, wound exudate and induration developed. A skin biopsy (1 × 1 × 0.2 cm) demonstrated a giant cell reaction and chronic inflammation consistent with a foreign body response; ampicillin/sulbactam was prescribed for 14 days. Approximately 8 months after the initial surgery, the right implant was removed because of patient-reported asymmetry; left breast revision was performed simultaneously. Intraoperative cultures yielded coagulase-negative *Staphylococcus* sensitive to ampicillin/sulbactam. A subsequent biopsy showed a chronic inflammatory process with abscess foci, giant cell reaction, and stromal fibrosis.

Approximately 12 months postoperatively, a cavity (1 × 0.7 cm) was noted in the lower pole with progressive deepening and clinical impression of rib involvement. The patient was referred to our center with a chronic left breast fistula and rib exposure. Imaging studies obtained at referral (plain chest X-ray and 3-phase bone scintigraphy) showed no evidence of active bone infection, rendering the diagnosis of rib osteomyelitis presumptive based on clinical and intraoperative findings. A multidisciplinary team comprising orthopedic surgery, plastic surgery, and infectious disease specialists assumed management.

**Figure 1.**
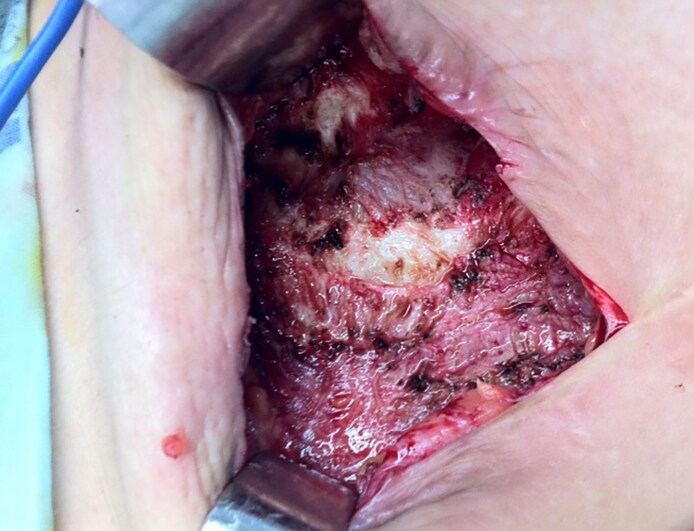
Intraoperative photograph of a 25-year-old female patient following surgical debridement and rib sequestrectomy, demonstrating exposure of the anterior rib surface with a 7 × 14 cm chest wall defect.

Approximately 16 months after the initial surgery, the first surgical intervention was performed: a 3- to 4 cm wound with rib exposure and a productive fistula were found. Debridement of the fistula tract, fibrotic tissue, and osteochondral surface was performed, yielding a 4 × 6 cm residual defect; vacuum-assisted closure (VAC) therapy was initiated, and cefalexin 500 mg every 6 h for 10 days was prescribed. Two days later, a second debridement revealed purulent secretion at the costochondral junction, requiring pectoralis major dissection, expanding the final skin defect to 7 × 14 cm (Figure 1). Bacterial cultures from both interventions were negative.

**Figure 2.**
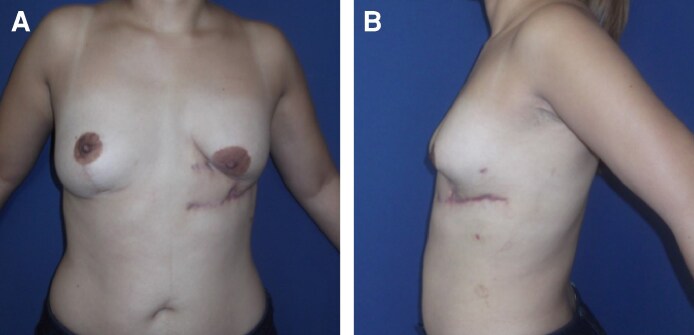
(A) Anterior view of a 26-year-old female patient at 6 months following chest wall reconstruction with an epigastric advancement-rotation flap, showing a residual contour defect of the left breast. (B) Left lateral view of the same 26-year-old female patient at 6 months following chest wall reconstruction with an epigastric advancement-rotation flap, demonstrating the residual left breast contour deformity.

**Figure 3.**
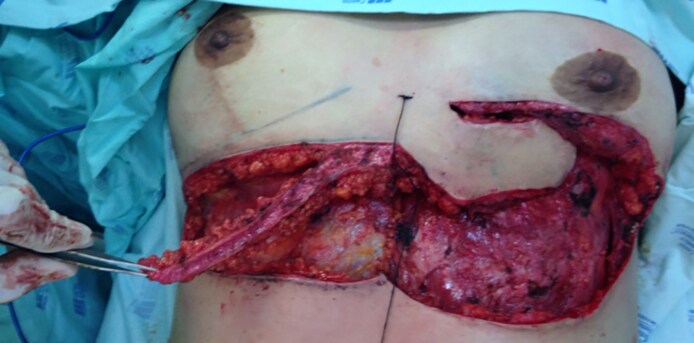
Intraoperative photograph of a 26-year-old female patient during definitive wound closure, showing the right fat flap used for inner inferior quadrant reconstruction and the left thoracoabdominal lipocutaneous pedicle flap to restore volume to the inner lower quadrant of the left breast.

Given negative bacterial cultures and the extent of the defect, an epigastric advancement-rotation flap was performed (Figure 2A, B). Subsequent wound dehiscence with serosanguineous secretion required further debridement and VAC therapy; a thoracoabdominal flap was then performed for definitive closure and breast volume restoration (Figure 3).

A further dehiscence episode prompted surgical debridement with repeat cultures. Empiric vancomycin (1 g IV every 12 h) and cefepime (1 g IV every 12 h) were initiated; HIV serology was negative. *Pseudomonas aeruginosa* (ciprofloxacin sensitive and piperacillin/tazobactam resistant) was isolated and treated with ciprofloxacin 400 mg IV every 8 h for 8 days, then 750 mg orally twice daily for 1 month. Mycobacterial cultures from left breast secretion and thoracic wall tissue (collected ∼1 year after implant removal following multiple debridements) identified *M. fortuitum*, sensitive to amikacin, clarithromycin, and doxycycline. The organism was isolated from wound tissue, not periprosthetic material. Clarithromycin 500 mg twice daily and moxifloxacin 400 mg once daily were administered for 6 months.

**Figure 4.**
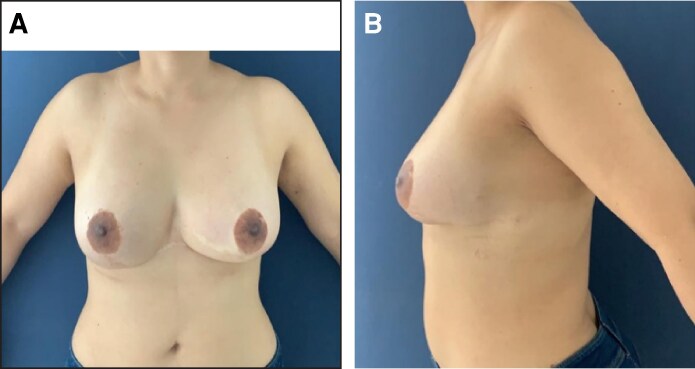
(A) Anterior view of a 29-year-old female patient at 4-year follow-up after staged management of chronic *Mycobacterium fortuitum* breast implant infection and bacterial superinfection, demonstrating satisfactory aesthetic outcome following bilateral autologous fat grafting. (B) Left lateral view of the same 29-year-old female patient at 4-year follow-up after staged management of chronic *M. fortuitum* breast implant infection and bacterial superinfection, demonstrating the final breast contour result.

Approximately 2 years after the culture-positive surgery, scar resection, flap remodeling, and bilateral autologous fat grafting were performed to address residual asymmetry, including 2 sessions to the right breast. At the 8-year follow-up, the patient reported satisfaction with her aesthetic outcome (Figure 4).

## DISCUSSION

*Mycobacterium fortuitum* breast implant infection is rare and often underdiagnosed because routine bacterial cultures return negative and mycobacterial-specific media are seldom employed. Al-Halabi et al reported *M. fortuitum* as the most frequently isolated NTM species in breast implant infections, accounting for 52.6% of mycobacterial cases.^9^ Macadam et al estimated NTM incidence at 0.013% of breast augmentation procedures but noted this is likely an underestimate.^16^ Our patient had no identifiable risk factors, consistent with the literature showing that NTM breast infections predominantly affect immunocompetent individuals.^9^

Hospital tap water represents the most plausible environmental reservoir. Jaubert et al demonstrated a direct link between postoperative *M. fortuitum* breast infection and hospital water supply through molecular typing, supporting the hypothesis that waterborne contamination during surgery is a key transmission route.^11^ Biofilm formation on implant surfaces enables microbial persistence in the face of standard antiseptics and antibiotics.^8^

The diagnosis of rib osteomyelitis in this case was presumptive: imaging studies were negative, and definitive microbiological confirmation was obtained from wound secretion rather than bone tissue, collected ∼1 year after implant removal and multiple debridements. Fewer than 7 cases of rib osteomyelitis complicating breast implant infection have been reported, all from Europe and Australia.^11-15^ When NTM infection is suspected clinically, specimens should be processed for acid-fast bacilli smear and culture on dedicated media (MGIT, Löwenstein–Jensen) with incubation ≥6 weeks; molecular techniques (polymerase chain reaction or 16S rRNA gene sequencing) can accelerate species identification.^8^

Treatment of *M. fortuitum* infection requires combined surgical and antibiotic management. Gonzalez-Santiago and Drage recommend 2 agent-directed oral therapies for a minimum of 4 months; clarithromycin or azithromycin combined with ciprofloxacin, doxycycline, or trimethoprim-sulfamethoxazole is preferred.^8^ Our 6-month regimen of clarithromycin and moxifloxacin achieved resolution.

Regarding prevention, povidone-iodine–based breast pocket irrigation has demonstrated in vitro bactericidal activity specifically against *M. fortuitum* and other resistant organisms.^17-21^ Dang et al and Jewell et al further support the use of antiseptic irrigation to reduce the microbial load in the implant pocket before closure.^20,21^ Although irrigation status at the index surgery could not be confirmed in this case, current evidence supports its routine use in implant-based breast surgery.^22-24^

## CONCLUSIONS

Rib osteomyelitis secondary to *M. fortuitum* breast implant infection is exceptionally rare. This case (the first reported in Latin America) demonstrates that the complication can occur in immunocompetent patients without identifiable predisposing factors. Clinical suspicion for NTM infection must be maintained when a postimplant wound fails conventional treatment and routine bacterial cultures are negative. Thorough serial surgical debridement with VAC therapy, prolonged susceptibility-guided antibiotic therapy, staged reconstructive surgery, and multidisciplinary management are essential for successful outcomes.
